# Semaglutide and Cardiovascular Outcomes According to Baseline Aspirin Use: Analyses of the SOUL and SELECT Trials

**DOI:** 10.1111/dom.70752

**Published:** 2026-04-13

**Authors:** Dirk Müller‐Wieland, Darren K. McGuire, A. Michael Lincoff, John B. Buse, Johannes F. E. Mann, Silvio E. Inzucchi, Rodica Pop‐Busui, Sharon L. Mulvagh, Scott S. Emerson, Neil R. Poulter, John E. Deanfield, Nikolaus Marx

**Affiliations:** ^1^ Department of Internal Medicine I RWTH Aachen University, University Hospital Aachen Aachen Germany; ^2^ Clinic for Cardiology, Angiology, and Intensive Care Medicine University of Texas Southwestern Medical Center, and Parkland Health System Dallas Texas USA; ^3^ Department of Cardiovascular Medicine Cleveland Clinic Lerner College of Medicine of Case Western Reserve University Cleveland Ohio USA; ^4^ University of North Carolina School of Medicine Chapel Hill North Carolina USA; ^5^ KfH Kidney Center Munich Germany; ^6^ Friedrich Alexander University of Erlangen Erlangen Germany; ^7^ Section of Endocrinology Yale University School of Medicine New Haven Connecticut USA; ^8^ Division of Endocrinology, Diabetes and Clinical Nutrition, Department of Medicine Oregon Health and Science University Portland Oregon USA; ^9^ Department of Medicine, Division of Cardiology Dalhousie University Halifax Nova Scotia Canada; ^10^ Department of Biostatistics University of Washington Seattle Washington USA; ^11^ Imperial Clinical Trials Unit Imperial College London London UK; ^12^ Institute of Cardiovascular Sciences University College London London UK

**Keywords:** antidiabetic drug, cardiovascular disease, clinical trial, GLP‐1 analogue

## 1 Background

Results from large cardiovascular outcome trials (CVOTs) in individuals with type 2 diabetes (T2DM) or overweight/obesity without diabetes at high CV risk have demonstrated that selected GLP‐1 receptor agonists (GLP‐1 RA) reduce cardiovascular events compared with placebo. The results of these trials have led to changes in guideline recommendations for the treatment of such patients with GLP‐1 RA [1]. A recently published real‐world data observational study from Lin et al., however, suggested that the benefits of GLP‐1 RAs may be attenuated or even reversed by in the presence of concomitant aspirin therapy. Results from their propensity‐score‐matched cohort analyses of administrative data comprising 19 022 matched individuals with obesity with or without T2DM, a subset out of 2.9 million meeting study eligibility criteria, found that the combination of GLP‐1 RA with aspirin was associated with higher CV risk compared with GLP‐1 RA in the absence of concomitant aspirin use [2]. To further explore this concern, we examined the effect of semaglutide on CV outcomes in participants in the randomized, placebo‐controlled SOUL and SELECT trials stratified by baseline use of aspirin.

## 2 Methods

We analysed the effect of semaglutide on key CV outcomes in participants in the SOUL and the SELECT trials stratified by baseline use of aspirin and by interaction testing for differential treatment efficacy of semaglutide. In the SOUL‐Trial, a CVOT in individuals with T2DM and established atherosclerotic CV disease (ASCVD) and/or chronic kidney disease (CKD), oral semaglutide (14 mg OD) significantly reduced the composite outcome of cardiovascular death, nonfatal myocardial infarction or nonfatal stroke (3P‐MACE) by 14% compared with placebo [3]. Similarly, in the SELECT trial of individuals with ASCVD and a body mass index of ≥ 27 kg/m2 but without diabetes, treatment with semaglutide (2.4 mg OW sc) significantly reduced the risk of 3P‐MACE by 20% compared with placebo [4]. No adjustments for multiplicity were performed.

## 3 Results

In SOUL, 68% (6517/9650) of participants were receiving aspirin at baseline, with similar rates across treatment groups: 66.9% (3326/4825) for semaglutide and 68.2% (3291/4825) for placebo. Participants on aspirin were younger (median: 66 vs. 68 years), less often female (25.9% vs. 35.1%), had a lower LDL cholesterol (median: 69.9 vs. 74.9 mg/dL), and more often had coronary heart disease (81.7% vs. 49.7%) compared with those not treated with aspirin. There were 395/3226 (semaglutide) versus 448/3291 (placebo) primary outcome events (HR 0.88; 95% CI, 0.77; 1.01) in the subgroup of aspirin users and 184/1599 versus 220/1534, respectively, in the subgroup of non‐aspirin users (HR 0.78; 95% CI, 0.64; 0.95; P‐interaction 0.33).

**FIGURE 1:**
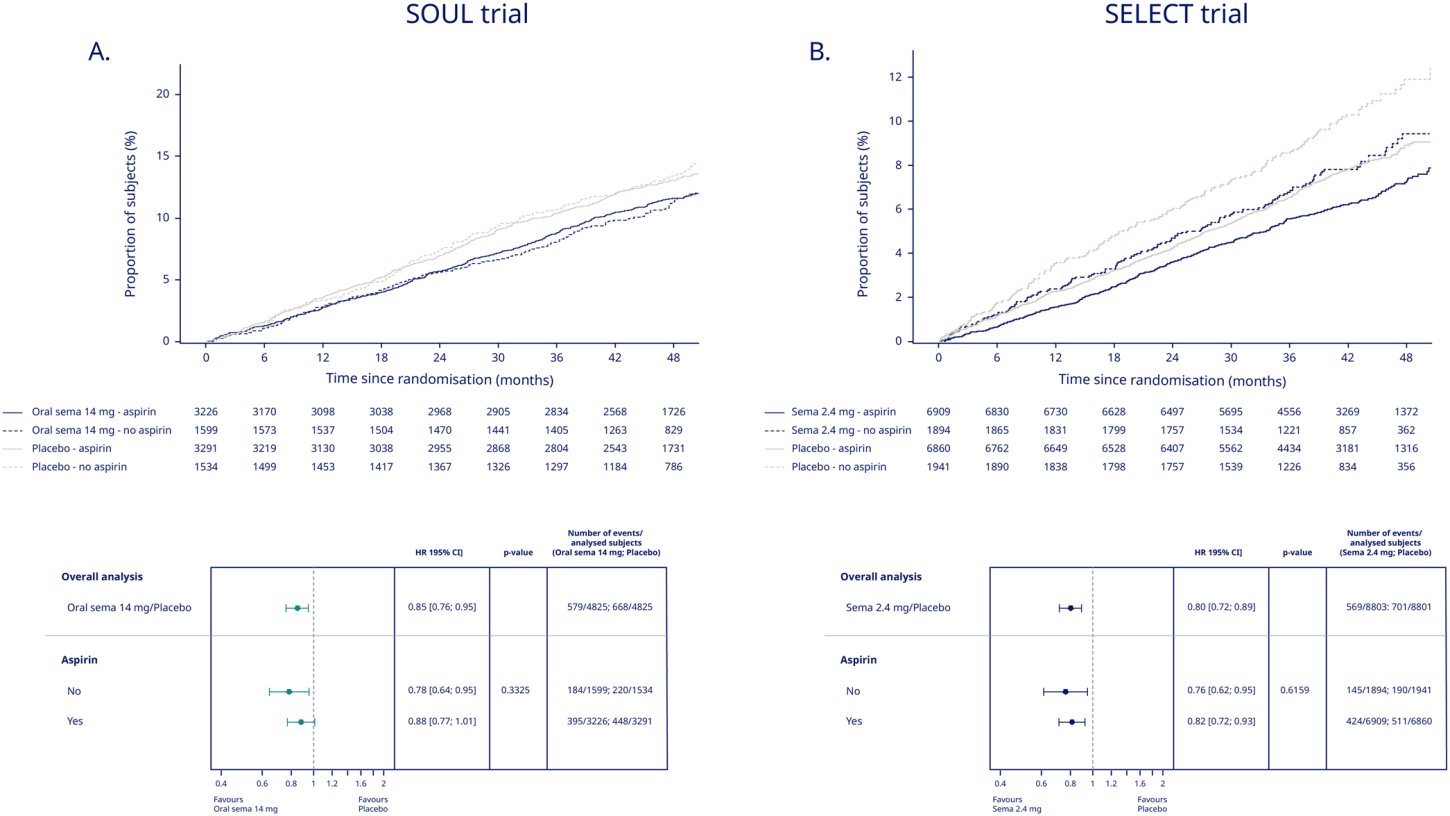
Cumulative incidence plots of time to first occurrence of MACE (CV death, non‐fatal MI, or non‐fatal stroke) from the SOUL trial (A, upper panel) and for SELECT (B, upper panel) for semaglutide versus placebo in subgroups with/without aspirin use at baseline. Lower panels show Forest plots: Time to first occurrence of primary outcomes according to Aspirin use at baseline in both trials. The cumulative incidence rate is calculated using Aalen‐Johansen method with all‐cause death as a competing risk. Data from the in‐trial period. For the overall analyses, estimated hazard ratio and corresponding confidence interval are calculated in a Cox proportional hazards model with treatment as a fixed factor. For the subgroup analyses, estimated hazard ratios and corresponding confidence intervals are calculated in a Cox proportional hazards model with treatment, subgroup and the interaction between treatment and subgroup as fixed factors. CI, confidence interval; EAC, event adjudication committee; HR, hazard ratio; MACE, major adverse cardiovascular event; *p* value, *p* value for test of the interaction effect.

In SELECT, 78% (13 769/17 604) of participants used aspirin at baseline, again balanced across treatment groups: 78.5% (6909/8803) for semaglutide and 77.9% (6860/8801) for placebo; they were also younger (median: 61 vs. 63 years), less often female (25.1 vs. 36.9%), had a lower LDL‐Cholesterol (median: 76.4 vs. 84.9 mg/dL), and more often had coronary heart disease (87.9% vs. 61.3%) but less frequently prior stroke (12.4% vs. 38.8%) compared with those not treated with aspirin. In aspirin users there were 424/6909 primary outcome events in the semaglutide group versus 511/6860 in the placebo group (HR 0.82; 95% CI, 0.72; 0.93); in non‐aspirin users the respective numbers were 145/1894 in the semaglutide and 190/1941 in the placebo group (HR 0.76; 95% CI, 0.62; 0.95; P‐interaction 0.62) (Figure 1).

## 4 Discussion

These data from 2 large CVOTs in individuals with T2DM and ACSVD and/or CKD (SOUL) or individuals with overweight/obesity without diabetes and ASCVD (SELECT) demonstrate no evidence of differential treatment effects of the GLP‐1 RA semaglutide on MACE outcomes according to baseline aspirin use. Compared to the matched samples used in the real‐world data observational analyses, the patient populations in the CVOTs were by design at higher risk of MACE: they tended to be older, more male, and with higher prevalence of prior MACE. The difference between the results from our analyses and the observational study by Lin et al. might stem from the known limitations of non‐randomized observational studies. Real‐world data provide important information on effectiveness and safety during routine care and complement data from randomized controlled trials (RCTs) [5] but their limitations include residual confounding, immortal time bias, study duration, incomplete ascertainment of drug exposure and outcome events. In the paper by Lin et al., after propensity score matching, data from only 19 022 patients of more than 2.9 million meeting study eligibility criteria were analysed. The propensity score method (PSM) is an approach typically used for evaluating non‐randomized therapy studies. However, PSM can only adjust for measured confounding variables, and thus confounding by indication remains a concern. A balanced distribution of unknown confounding variables can be assured only through randomization, and it is for this reason that RCTs are considered the gold standard to address important questions regarding the efficacy of CV treatment strategies. A further limitation of the Lin study is that the dataset employed is largely comprised of aggregated electronic health record data. Using structured data alone, a prior report suggested that assignment of exposure could not be performed with high confidence, even in a single electronic health record [6].

Our study has limitations: these were exploratory analyses not prespecified in the statistical analysis plans; neither of the trials was powered to assess for differential treatment effects of semaglutide for CV outcomes with or without background aspirin use, and randomization of participants in both trials was not stratified according to aspirin use. Randomization is expected to preserve population characteristics between treatment groups. Of note, this is not the case when comparing aspirin use (yes vs. no) within treatment groups, as baseline aspirin use was not randomized. Consequently, differences in baseline characteristics between aspirin users and non‐users within the same treatment group and thus in the whole population are expected. Despite these limitations, the present results deriving from RCTs where data on background medical therapy were systematically collected and events of interest were prospectively assessed and centrally adjudicated provide assurance for clinicians that semaglutide reduces CV events in a broad population of high‐risk individuals regardless of concomitant aspirin use.

In conclusion, the current analyses from the SOUL and SELECT trials suggest that semaglutide significantly reduces major adverse cardiovascular events, irrespective of baseline aspirin therapy in individuals with T2DM or overweight/obesity without diabetes at high CV risk.

## Funding

The SOUL trial was funded by Novo Nordisk A/S. The funder of the study was responsible, along with an academic steering committee, for the study design, contributed to data collection, data analyses, and data interpretation, and participated in the review of the manuscript in collaboration with the authors.

## Ethics Statement

National and institutional regulatory and ethical authorities approved the protocols.

## Consent

All participants provided written informed consent for research participation.

## Conflicts of Interest

The statistical analyses were performed by Søren Rasmussen, PhD; MSc, who is employed by and holds stocks in Novo Nordisk. Nikolaus Marx, M.D. is supported by the Deutsche Forschungsgemeinschaft (German Research Foundation; TRR 219; Project‐ID 322900939 [M03, M05]). N.M. has given lectures for Bayer, Boehringer Ingelheim, Sanofi‐Aventis, MSD, BMS, AstraZeneca, Lilly, Novo Nordisk; has received unrestricted research grants from Boehringer Ingelheim, and has served as an advisor for Bayer, Boehringer Ingelheim, Sanofi‐Aventis, MSD, BMS, AstraZeneca, Novo Nordisk. In addition, he served in trial leadership for Boehringer Ingelheim and Novo Nordisk. N.M. declines all personal compensation from pharma or device companies. Dirk Müller‐Wieland, M.D. reports receiving consulting fees and speaker honoraria from Amarin, Amgen, AstraZeneca, Bayer, Boehringer Ingelheim, Daiichi‐Sankyo, GlaxoSmithKline, Lilly, Merck Sharp & Dohme, Novo Nordisk, and Sanofi. A. Michael Lincoff, MD reports receiving consulting fees from Novo Nordisk, Eli Lilly, Alnylam, Amgen, Ardelyx, Brainstorm Cell, Canary Cure, Capricor, Cadrenal, Johnson & Johnson, Medtronic, Neovasc, ReCor, TD Cowen, and V‐Wave and research funding to his institution from AbbVie, CSL Behring, Eli Lilly, Esperion, Novartis. John E. Deanfield, M.D. reports grants or contracts 2022–2025 from Alzheimer's Research UK and 2019–2022 from British Heart Foundation. He received consulting fees/honoraria from Amgen, AstraZeneca, Boehringer Ingelheim, Merck, Pfizer, Aegerion, Novartis, Sanofi, Takeda, Novo Nordisk, and Bayer. Johannes F.E. Mann, M.D. reports grants from Novo Nordisk, the European Union and McMaster University Hamilton, Canada; consulting fees from Novo Nordisk, AstraZeneca, Bayer and Boehringer Ingelheim; honoraria from Novo Nordisk, AstraZeneca, Bayer and Novartis; and has participated on a data safety monitoring board or advisory board for AstraZeneca, Bayer, Sanofi and Boehringer Ingelheim, as well as an author for UpToDate and a leadership role in the KDIGO group. John B. Buse, M.D. has received salary support from clinical trial contracts to his employer by Corcept, Dexcom, GentiBio, Novo Nordisk, and Sparrow Pharmaceuticals; has received salary support from consulting contracts to his employer by Novo Nordisk; he is a consultant with personal compensation from Aardvark Therapeutics, Altimmune, Alveus Therapeutics, Amgen, Antag, Aqua Medical, AstraZeneca, Boehringer‐Ingelheim, Corcept Therapeutics, Dexcom, Eli Lilly, embecta, General Medicines Inc., GentiBio, Kayothera, Metsera, MiniMed, Pfizer, Recordati, Sparrow Pharmaceuticals, Vertex, vTv Therapeutics, and Zealand; he has stock/options in Aardvark, Glyscend, Mellitus Health, Metsera, Pendulum Therapeutics, Praetego, and Stability HealthScott S. Emerson, Ph.D. declares consulting fees from Novo Nordisk for Steering Committee participation and from Boehringer Ingelheim for consulting re veterinary CHF. He also received support for attending meetings and/or travel from Novo Nordisk for attendance at Steering Committee and scientific meetings. He declares participation on an advisory board for Novo Nordisk, AstraZeneca, Daiichi Sankyo, Vertex, Roche, GlaxoSmithKline, Lilly, Novartis, Bristol Myers and Sanofi. Silvio E. Inzucchi, M.D. has served as a consultant or on advisory boards for Novo Nordisk, AstraZeneca, Boehringer Ingelheim, Bayer, and Corcept. He has given lectures sponsored by AstraZeneca and Boehringer Ingelheim/Lilly. He declares royalties from McGraw Hill and Wolters Kluwer Health and support for attending meetings and/or travel from Novo Nordisk, AstraZeneca, Boehringer Ingelheim/Lilly, and Bayer. Sharon L. Mulvagh, M.D. has received consulting fees/honoraria for serving as a consultant and/or on advisory boards for Novo Nordisk and Merck. Rodica Pop‐Busui, M.D., Ph.D. received research grant support to her institutions from NIDDK, Breakthrough T1D (former JDRF), Bayer, Lexicon Pharmaceuticals and Novo Nordisk, and consulting fees from Averitas Pharma, Lexicon Pharmaceuticals, Nevro Inc., Novo Nordisk, Roche Diagnostic, Vertex; support for attending meetings and/or travel from Novo Nordisk and Roche; participation on an advisory board for Biogen/Reata and is a Member of the Board of Directors of the American Diabetes Association. Neil R. Poulter, F.Med.Sci. received financial support from several pharmaceutical companies which manufacture BP‐lowering, lipid lowering, and glucose‐lowering agents for consultancy fees, research projects, and staff and for arranging and speaking at educational meetings including Servier, Pfizer, AstraZeneca, Amgen, Sanofi, Novo Nordisk. He holds no stocks and shares in any such companies. Darren McGuire, M.D. has received consulting fees from Novo Nordisk, AstraZeneca, Pfizer, Altimmune, Ventyx Pharmaceuticals, Bayer, Lexicon, Applied Therapeutics, Intercept Pharmaceuticals, Esperion, Lilly USA, Boehringer Ingelheim, New Amsterdam, CSL Behring, Amgen, Neurotronics, Metsera, Kailera and Alveus Pharma. Research funding to his institution from AbbVie, CSL Behring, Eli Lilly, Esperion, Novartis.

## Data Availability

An authorised researcher can request access to clinical trial data by submitting a research proposal for review and approval by Novo Nordisk and an internal independent review panel. Requests are usually considered after the research is finished and the main results have been published. If the research supports a regulatory application, requests will be considered after the product and its intended use are approved in both the EU and the USA. Participants clinical data will be anonymised, following an approved internal process, before data are shared to external third parties. For details on how to request access to clinical data, visit novonordisk‐trials.com.
